# Effect of advanced glycosylation end products on apoptosis in human adipose tissue-derived stem cells *in vitro*

**DOI:** 10.1186/2045-3701-5-3

**Published:** 2015-01-27

**Authors:** Zhe Wang, Hongqiu Li, Dianbao Zhang, Xiaoyu Liu, Feng Zhao, Xining Pang, Qiushi Wang

**Affiliations:** Department of Blood Transfusion, Shengjing Hospital of China Medical University, No. 36 Sanhao Street, Heping District, Shenyang, 110004 P. R. China; Department of Orthopaedics, Shengjing Hospital of China Medical University, No. 36 Sanhao Street, Heping District, Shenyang, 110004 P. R. China; Department of Orthopedics, Central Hospital of Shenyang Medical College, No.5 Nanqi Road, Tiexi District, Shenyang, 110024 P.R. China; Department of Stem Cells and Regenerative Medicine, Key Laboratory of Cell Biology, Ministry of Public Health and Key Laboratory of Medical Cell Biology, Ministry of Education, China Medical University, No. 92 Bei’er Road, Heping District, Shenyang, 110001 P. R. China

**Keywords:** Human adipose tissue-derived stem cells, Advanced glycation end products, Mitogen-activated protein kinase, Apoptosis, Receptor for advanced glycation end products

## Abstract

**Background:**

Both apoptosis and caspase-3 activity in adipose tissue-derived stem cells play an important role in the therapeutic process of diabetes patients. The purpose of this study was to investigate the effect of advanced glycation end products-human serum albumin (AGE-HSA) on apoptosis in human adipose tissue-derived stem cells (ADSCs) and to characterize the signal transduction pathways activated by AGEs that are involved in apoptosis regulation.

**Results:**

AGE-HSA promoted apoptosis and caspase-3 activity in ADSCs. However, the effects of AGE-HSA were significantly attenuated by an inhibitor of p38 MAPK, but not by inhibitors of JNK MAPK or ERK MAPK. AGE-HSA also upregulated the expression of RAGE. Silencing of the RAGE gene inhibited AGE-HSA-induced apoptosis, and activation and expression of phosphorylated p38 MAPK.

**Conclusions:**

These results suggest that AGE-HSA promote the apoptosis of ADSCs *in vitro* via a RAGE-dependent p38 MAPK pathway.

## Background

Advanced glycation end products (AGEs), which are produced by the posttranslational modification of proteins via non-enzymatic glycations, accumulate with age, and are abundantly increased in diabetic patients [1–3]. The formation and accumulation of AGEs are characteristic of tissues from patients with diabetes mellitus and have been strongly implicated in the pathogenesis of diabetic complications [4, 5]. AGEs are slowly and irreversibly formed on proteins that are exposed to carbonyl and substrate stress, especially under conditions of hyperglycemia, hyperlipidemia and/or oxidative stress [6]. AGEs play an important role in the pathophysiological processes affecting patients with diabetes, Alzheimer's disease, and aging [2, 7, 8]. AGEs are known to cause adverse side effects on the growth of several cells, such as vascular endothelial cell and renal tubular epithelial cell [9–17]. A number of studies have suggested that AGEs mediate cell apoptosis and may play an important role in the pathogenesis of biophysical disorders [4, 6]. The elevated AGEs in diabetic patients could induce a number of pathological changes, such as promoting endothelial progenitor cell (EPC) and endothelial cell apoptosis [9, 18].

The receptor for advanced glycation end products (RAGE) is a signal transduction receptor, which senses a variety of signaling molecules including advanced glycation end products (AGEs). RAGE is a 35 kDa multiligand transmembrane receptor which belongs to the immunoglobulin gene superfamily [19]. RAGE is widely localized in many cell types such as smooth muscle cells, hepatocytes, neurons, endothelial cells, and monocytes. Its expression can be abnormally up or downregulated in human diseases [20–22].RAGE is highly expressed in the bones of diabetic animal models compared to control animals [23]. Recently, adipose tissue-derived stem cells (ADSCs) have been suggested as ideal for cell therapy because of their simple isolation techniques, easy expandability, low immunogenicity, and pluripotency [24, 25]. ADSC therapy could potentially have a large impact on the treatment of a wide variety of diseases, including diabetes and diabetic complications. However, increased apoptosis in stem cells has been demonstrated in a diabetic rat model [26]. The deleterious effects of AGEs that involve RAGE expression were reported in stem cells [27].Nevertheless, there are few reports about the role of RAGE in ADSC therapy in complaint such as diabetes.

AGEs have been reported to activate serine-threonine protein kinases in the mitogen-activated protein kinase (MAPK) pathways. C jun N terminal kinase (JNK) and p38 MAPK are the two major subfamilies of MAPKs that participate in apoptosis. AGEs are known to induce osteoblast apoptosis via both JNK and p38 MAPK [15, 17, 27]. Despite ADSC administration was reported to improve healing in diabetic skin repair [28, 29], it remains unclear if AGEs could affect the apoptosis of ADSCs during the therapeutic process in diabetes patients. The purpose of the present study was to determine the effect of advanced glycation end products-human serum albumin (AGE-HSA) on ADSC apoptosis and to investigate the underlying molecular mechanism. The results of this study could lay a cellular basis for more effectively using ADSCs to treat diabetes and diabetic complications.

## Results

### Characterization of ADSCs

**Figure 1:**
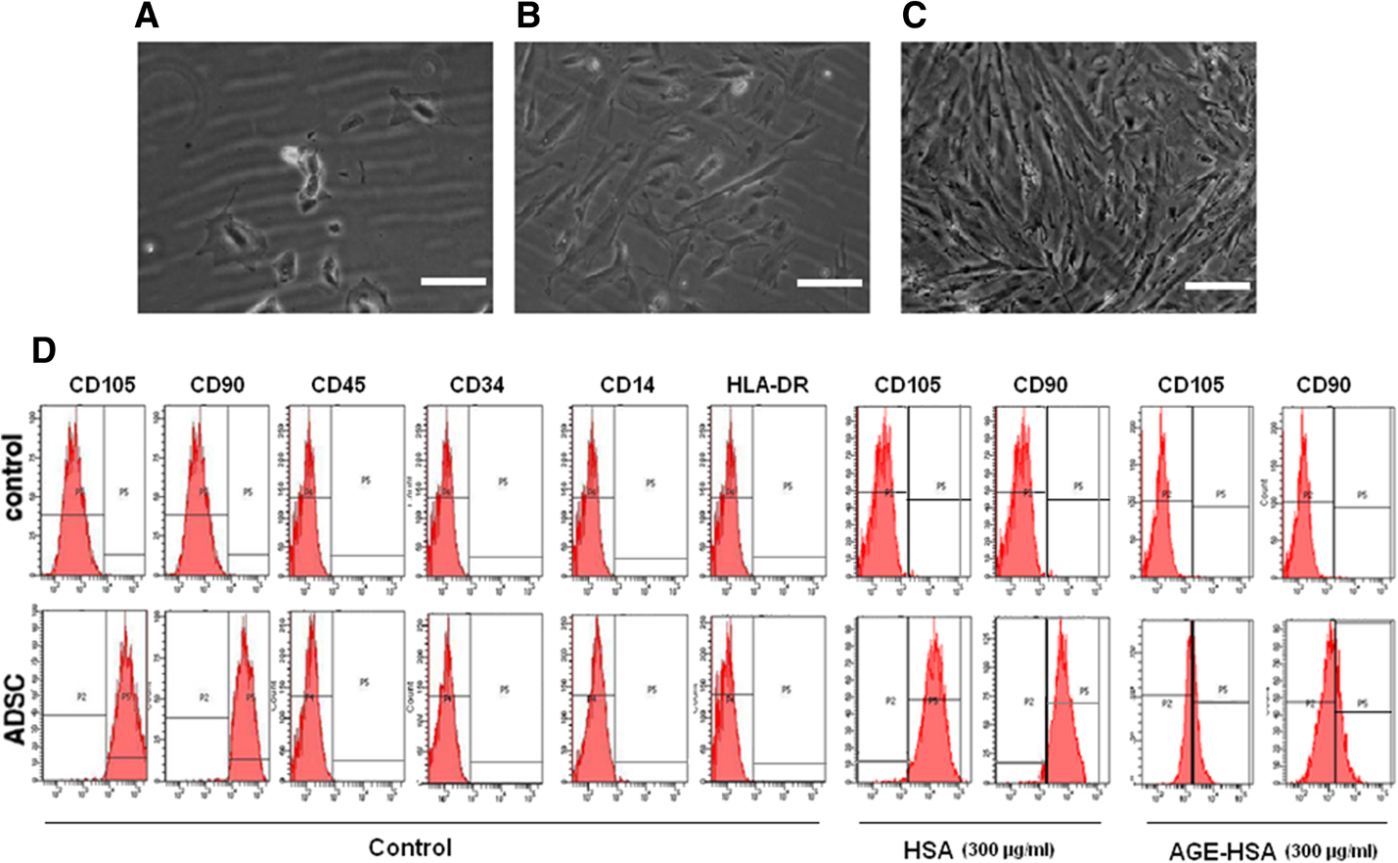
**Characterization of ADSCs and the effect of AGE-HSA on CD105 and CD90 expression in ADSCs. (A)** Representative image of isolated ADSCs on the first day. **(B)** Adherent ADSCs 3 days after plating, showing a spindle shape. **(C)** After 10 days, most of the ADSCs exhibited fibroblast-like morphologies and had reached near-confluence. Scale bar is equivalent to 50 μm. **(D)** Passage 3 ADSCs were labeled with antibodies or matching isotype controls against the indicated antigens and analyzed by flow cytometry to obtain representative immunophenotypes. FACS showed that ADSCs expressed CD105 and CD90, but not CD34, CD14, CD45 or HLA-DR. AGE-HSA altered CD105 and CD90 expression in ADSCs.

ADSCs were isolated from human adipose tissue. As shown in Figure 1, adherent cells were seen after 1 d in culture (Figure 1A). After 3–4 d in culture, the cells had a short spindle shape (Figure 1B). Most of the cells exhibited fibroblast-like morphologies and were nearly confluent after 10 d in culture (Figure 1C). To characterize the cell surface marker profile of stem cells from human adipose tissue, cells from the third passage were analyzed by FACS. FACS showed that ADSCs expressed CD105 and CD90, but not CD34, CD14, CD45 or HLA-DR, consistent with previous reports [10] (Figure 1D). The cells were incubated with HSA (300 μg/ml) or AGE-HSA (300 μg/ml) for 24 h. Cells treated with AGE-HSA showed an obvious decrease in the percentage of cells expressing the stem cell-specific markers (CD105 and CD90) (Figure 1D and Table 1).

**Table 1:** **Effects of AGE-HSA on cell surface antigen expression of ADSCs**

Marker	HSA (300 μg/ml)	AGE-HSA (300 μg/ml)
**CD105**	**97.5 ± 2.01**	39 ± 4.27*
**CD90**	**96.9 ± 1.95**	28.5 ± 6.14*

*p < 0.05 vs. control (HSA 300 μg/ml).

### Effects of AGE-HSA on apoptosis and caspase-3 activity of ADSCs

**Figure 2:**
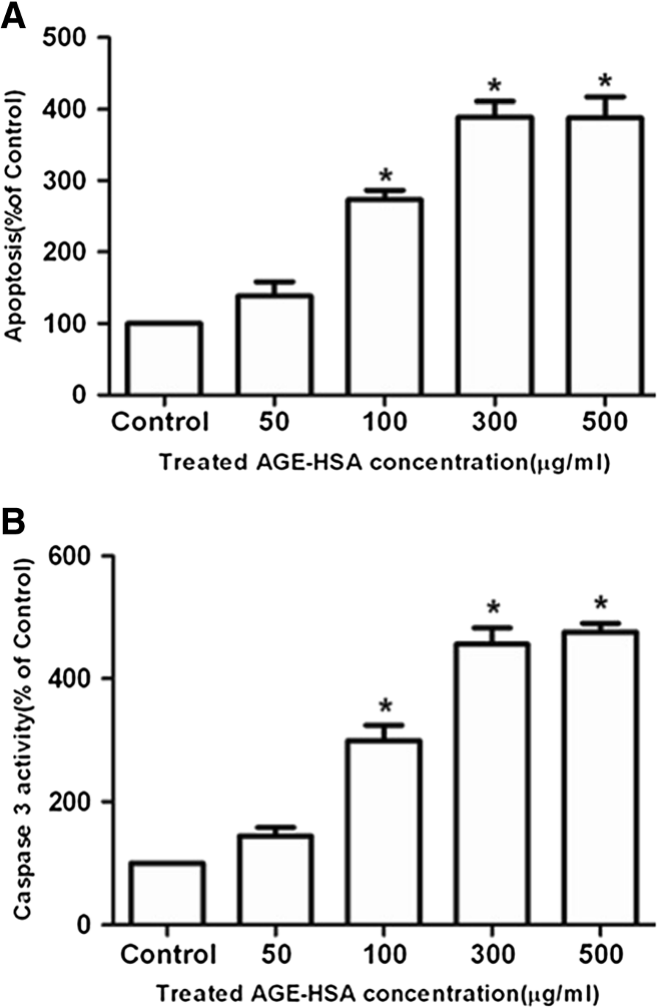
**The effect of AGE-HSA on apoptosis and caspase-3 activity in ADSCs.** The cells were incubated with HSA (300 μg/ml) or AGE-HSA (50–500 μg/ml) for 24 h. The level of apoptosis **(A)** and caspase-3 activity assay **(B)** were measured by ELISA. Each value is expressed as the mean ± SD of three independent experiments. *p < 0.05 vs. control (HSA 300 μg/ml).

The cells were incubated with HSA (300 μg/ml) or AGE-HSA at concentrations ranging from 50–500 μg/ml for 24 h. As shown in Figure 2, the cells treated with AGE-HSA showed an increase in apoptotic cell death compared to control cells (Figure 2A). Because caspase-3 is the principal effector caspase through which the mitochondrial and cytosolic pathways induce apoptosis, the levels of caspase-3 activity in each group were measured (Figure 2B). AGE-HSA significantly increased the caspase-3 activity in treated cells compared to control cells.

### Effects of AGE-HSA on expression of RAGE of ADSCs

**Figure 3:**
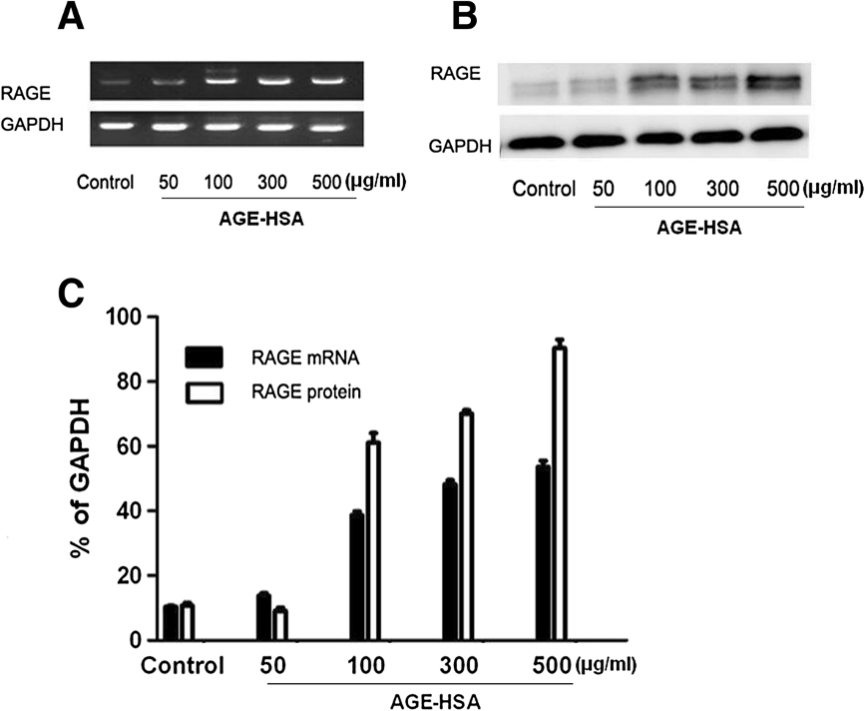
**The effect of AGE-HSA on expression of RAGE. (A)** Representative RT-PCR images showing mRNA expression levels of RAGE. **(B)** Representative Western blot images showing the protein expression levels of RAGE. **(C)** Densitometric analyses of RT-PCR and Western blots as a percentage of GAPDH expression. Each value is expressed as the mean ± SD of three independent experiments. *p < 0.05 vs. control (HSA 300 μg/ml).

The effects of AGEs are mediated by interaction with or uptake by RAGE. To determine whether AGE-HSA might affect the expression of RAGE in ADSCs, the cells were incubated with HSA (300 μg/ml) or AGE-HSA (50–500 μg/ml) for 24 h, and then the expression of RAGE was determined by RT-PCR and western blot. As shown in Figure 3, cells treated with AGE-HSA showed an increase in expression of RAGE mRNA levels (Figure 3A and C) and protein levels (Figure 3B and C).

### Role of MAPK pathway in AGE-HSA-promoted apoptosis of ADSCs

**Figure 4:**
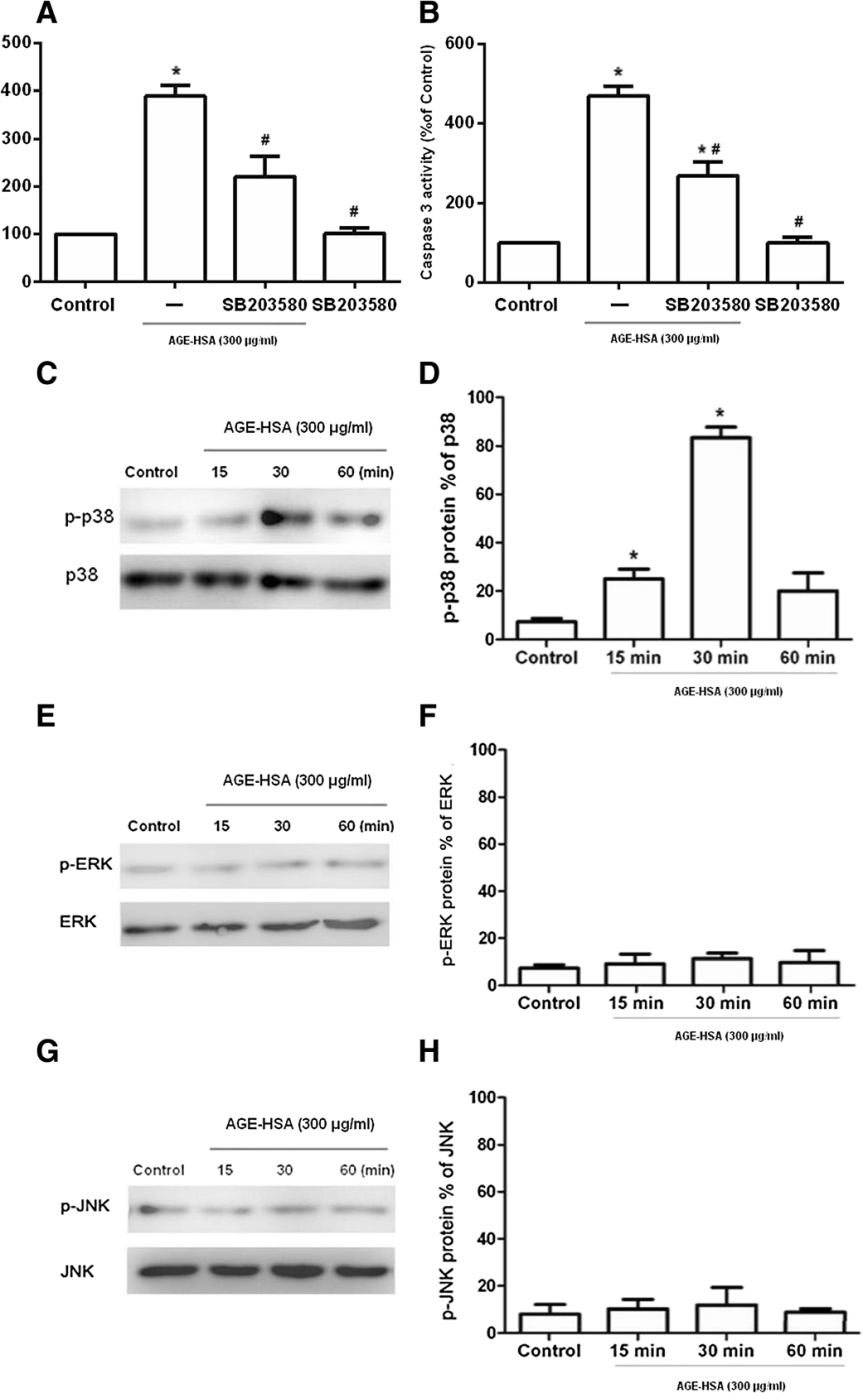
**Role of the MAPK pathway in AGE-HSA induced apoptosis in ADSCs.** The effect of a p38 MAPK inhibitor on apoptosis and caspase-3 activity in ADSCs induced by AGE-HSA. **(A, B)** Pre-treatment with a p38 inhibitor (SB203580) significantly reduced AGE-HSA induced increase in apoptosis and caspase-3 activity. **(C,E, G)** Representative western blot images showing protein expression of phosphorylated p38, ERK and JNK (p-p38, p-ERK and p-JNK). **(D, F, H)** Densitometric analyses of Western blots showing the level of p-p38, p-ERK and p-JNK as a percentage of total p38, ERK and JNK. Each value is expressed as the mean ± SD of three independent experiments. *p < 0.05 vs. control (HSA 300 μg/ml); # p < 0.05 vs. AGE-HSA group.

To determine whether the activation of MAPK was necessary for AGE-HSA-induced apoptosis in ADSCs, the cells were pre-treated for 60 min with pharmacological inhibitors of MAPK signaling pathways (U0126, SB203580, and SP600125). The cells were then treated with 300 μg/ml AGE-HSA for 24 h. Pre-treatment with the p38 inhibitor (SB203580) significantly reduced the AGE-HSA-mediated increase in apoptosis (Figure 4A) and caspase-3 activity (Figure 4B). Compared with the control group, SB203580 alone did not affect apoptosis (Figure 4A) and caspase-3 activity (Figure 4B). In contrast, U0126 (ERK) and SP600125 (JNK) had no effect on the level of apoptosis and caspase-3 activity induced by AGE-HSA. To further establish whether p38 MAPK phosphorylation was required for AGE-HSA induced apoptosis, cells were serum-starved and treated with AGE-HSA then analyzed for ERK1⁄2, p38, and JNK MAPK phosphorylation levels. p38 phosphorylation was rapidly induced in response to AGE-HSA. The peak response was obtained within 30 min and then phosphorylation declined to almost the basal level within 60 min. (Figure 4C-D). In contrast, AGE-HSA had no effect on activation of ERK1/2 and JNK MAPK (Figure 4E-H). These data indicated that the activation of p38 MAPK, but not the ERK1⁄2 and JNK pathways, was involved in AGE-HSA-induced apoptosis in ADSCs.

### Effects of RAGE siRNA on apoptosis and caspase-3 activity of ADSCs induced by AGE-HSA

**Figure 5:**
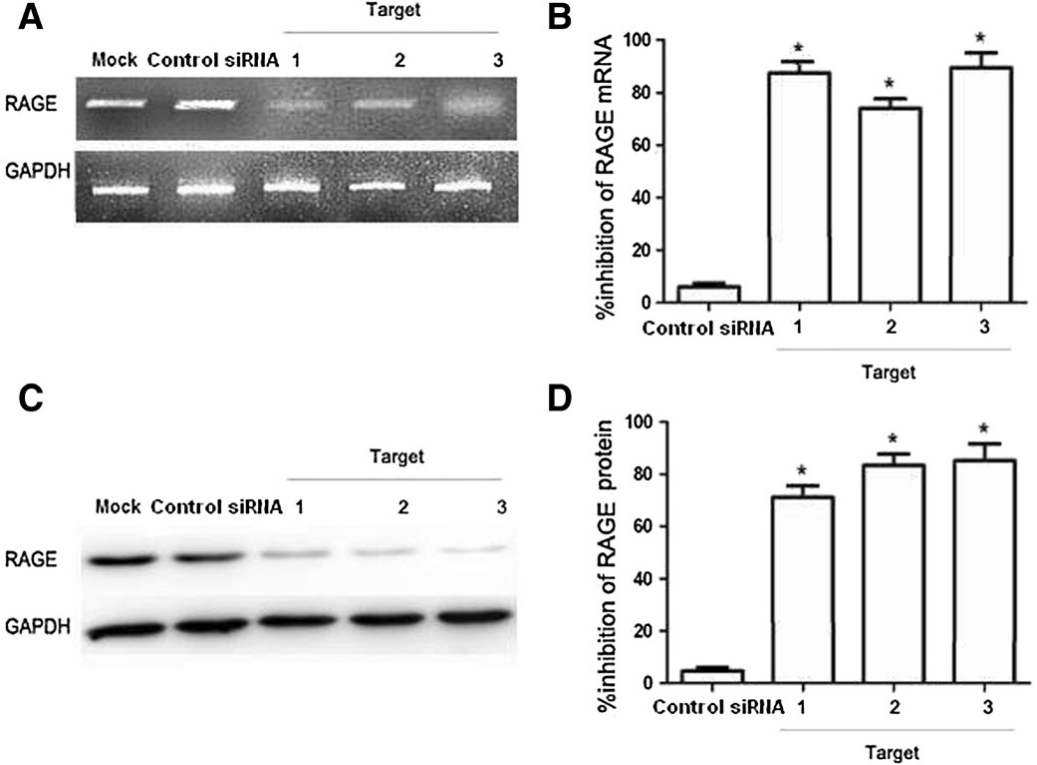
**Knockdown of RAGE mRNA and protein expression levels using siRNA.** Serum-starved ADSCs were transfected with different double-stranded siRNAs at a final concentration of 50 nM each. RAGE mRNA and protein expression levels at 48 h after transfection were quantified. **(A)** Representative RT-PCR experiments illustrating RAGE mRNA expression in ADSCs at 48 h after transfection with different siRNAs. **(B)** Data shown are the percent inhibition of RAGE mRNA expression levels compared to mock-transfected cells. The mean ± SD of three independent experiments are shown. **(C)** Representative Western blotting experiment illustrating RAGE protein expression in ADSCs 48 h after transfection with different siRNAs. **(D)** Data shown are the percent inhibition of RAGE protein expression in ADSCs 48 h after transfection with different siRNAs. The means ± SD of three independent experiments are shown. “Mock” indicates “mock-transfected cells”, meaning cells were only transfected with transfection reagents. *p < 0.05 vs. control siRNAs.

To test whether siRNA could silence RAGE expression, we treated ADSCs with three different RAGE siRNAs. After 24 h, the percentages of inhibition for RAGE mRNA expression were 87.3% (Target 1), 72.9% (Target 2), and 90.9% (Target 3) for each of the different siRNAs (Figure 5B) RAGE mRNA downregulation was paralleled by a decrease in RAGE protein abundance. The percentages of inhibition for RAGE protein expression were 71.5% (Target 1), 81.7% (Target 2), and 89.1% (Target 3) at 24 h (Figure 5C). Because Target 3 appeared to be the most effective at inhibiting RAGE mRNA and protein expression, this siRNA was used for subsequent experiments.

**Figure 6:**
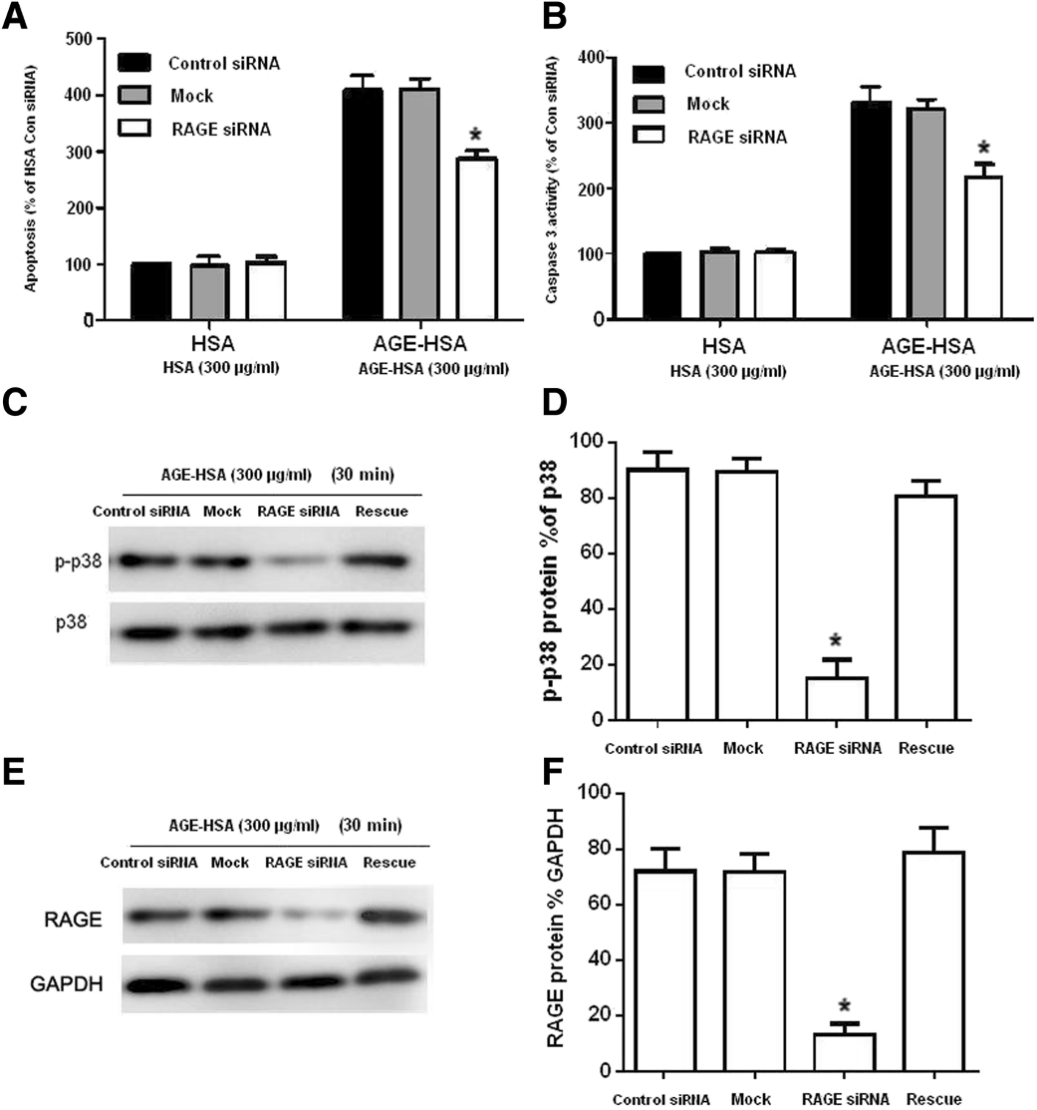
**Effects of RAGE siRNA on p38 MAPK activation in ADSCs induced by AGE-HSA. (A, B)** The mock-, control siRNA- and RAGE siRNA-transfected ADSCs were cultured with 300 μg/ml AGE-HSA or HSA for 24 h and then subjected to an ELISA to measure apoptosis and caspse-3 activity. **(C)** The mock-, con siRNA- and RAGE siRNA-transfected ADSCs were stimulated with 300 μg/ml AGE–HSA for 30 min. Phosphorylation of p38 MAPK was determined by Western blotting. Representative western blot images showing the protein expression of total p38 MAPK and phosphorylated p38 MAPK (p-p38) are shown. **(D)** Densitometric analyses of the western blots showing the level of p-p38 as a percentage of total p38. Each value is expressed as the mean ± SD of three independent experiments. “Mock” indicates “mock-transfected cells”, meaning cells were only transfected with transfection reagents. “Rescue” indicates cells in rescue condition (RAGE siRNA + RAGE expression). **(E)** The mock-, con siRNA- and RAGE siRNA-transfected ADSCs were stimulated with 300 μg/ml AGE–HSA for 30 min. Expression of RAGE was determined by Western blotting. Representative western blot images showing the protein expression of RAGE and GAPDH are shown. **(F)** Densitometric analyses of the western blots showing the level of RAGE as a percentage of GAPDH. Each value is expressed as the mean ± SD of three independent experiments. *p < 0.05 vs. control siRNA.

We next evaluated whether silencing the RAGE gene might affect the level of apoptosis and caspase-3 activity of ADSCs induced by AGE-HSA. The mock-, control siRNA- or RAGE siRNA-transfected ADSCs were supplemented with either HSA or AGE-HSA (300 μg/ml) for 24 h. The level of apoptosis and caspase-3 activity was then quantified by ELISA. No significant differences were observed in the level of apoptosis or caspase-3 activity between the three groups in the cells treated with HSA. However, after incubation with AGE-HSA, the level of apoptosis (Figure 6A) and caspase-3 activity (Figure 6B) was partly inhibited by the RAGE-specific siRNA compared to the control siRNA. In the groups that were mock transfected or transfected with control siRNA, the levels of apoptosis and caspase-3 activity were not significantly different.

### Effects of RAGE siRNA on p38 MAPK activation of ADSCs induced by AGE-HSA

To determine whether silencing the RAGE gene affected AGE-HSA mediated signaling downstream in the MAPK pathway, we evaluated p38 MAPK phosphorylation in the mock-, control siRNA- RAGE siRNA-, or RAGE siRNA + *PCDNA3.1-RAGE* expression construct (rescue) transfected ADSCs. The cells were supplemented with either HSA or AGE-HSA (300 μg/ml) for 24 h. Then, p38 MAPK phosphorylation and RAGE was quantified by Western blot analysis. No significant differences in the phosphorylation of p38 were observed between the three groups treated with HSA. However, after incubation with AGE-HSA, the phosphorylation of p38 was partly inhibited by the RAGE-specific siRNA compared with the non-binding control siRNA (Figure 6C-D). In groups that were mock transfected, transfected with control siRNA or in rescue condition, the p38 phosphorylation was not significantly different. Moreover, the expression of RAGE in these cells was also quantified by Western blot analysis (Figure 6E-F).

## Discussion

ADSCs have similar features to MSCs from other tissues such as a high proliferative rate and the potential to differentiate into diverse cell lineages of both mesodermal and nonmesodermal origin. Moreover, ADSCs are also abundant and easy to sample in adults, which could potentially allow them to be used for autologous transplantation [24–27]. Recent preclinical studies have shown the beneficial effect of ADSC administration for treating a wide variety of diseases, including in animal models of diabetes [28–31]. However, the expansion and differentiation of ADSCs *in vitro* could be affected by many factors including: growth factors, chemical signals, and seeding density that may all indirectly influence the subsequent therapeutic effects. In addition, the culture media components may influence stem cell proliferation replicative senescence, and apoptosis [26].

AGEs have been shown to stimulate the activation of MAPK cascades in different cell types [11–15]. Furthermore, MAPK signals are robustly activated in a variety of disease states and have been implicated in mediating apoptotic responses. AGEs have been reported to induce apoptosis in osteoblasts and fibroblasts via the JNK and p38 MAPK pathways [16, 17]. Based on these data, we hypothesized that AGE-HSA induced apoptosis in ADSCs could involve the MAPK pathways. Thus, we investigated the role of p38, ERK1⁄2, and JNK MAPK signaling in apoptosis and caspase-3 activity in ADSCs. Our data showed that AGE-HSA induced the phosphorylation of p38 MAPK, and that pretreatment with SB203580 inhibited AGE-HSA-induced apoptosis, suggesting that p38 MAPK potentially played an important role in regulating AGE-HSA induced apoptosis. In contrast, specific inhibitors of ERK and JNK, had no effect on the level of apoptosis in ADSCs.

RAGE is the best-characterized AGE receptor and is responsible for most of the damaging effects of AGEs [32–34]. Here, we demonstrated that ADSCs expressed RAGE protein and that the incubation of ADSCs with AGE-HSA resulted in significant upregulation of RAGE expression. Our results were consistent with Kume et al., who showed that MSCs expressed RAGE, and that its induction was stimulated by AGE-2 and AGE-3. Previous reports have shown that downstream apoptotic signals from RAGE can be mediated through the p38 MAPK and JNK pathways. In osteoblast cells CML-collagen-induced apoptosis, and therefore impaired bone formation, was reduced by p38 MAPK (45%) or JNK (59%) inhibitors, and the effect was additive as treatment with both kinase inhibitors caused a 90% reduction in cell apoptosis [21]. Furthermore, AGE-mediated apoptosis in endothelial progenitor cells was shown to be significantly inhibited by anti-RAGE neutralizing antibody [35]. To confirm the involvement of RAGE in mediating apoptosis by AGE-HSA, we used an siRNA approach to block RAGE in ADSCs. We found that siRNAs significantly suppressed AGE-HSA stimulated apoptosis. These results demonstrate the critical role of RAGE in mediating stem cell survival and highlight the importance of the RAGE ligand axis in ADSC therapy for diabetes. Furthermore, knocking down RAGE expression resulted in an obvious decrease in the level of p38 MAPK phosphorylation stimulated by AGE-HSA. This suggests that the activation of p38 MAPK stimulated by AGE-HSA might be RAGE dependent.

## Conclusion

The present study demonstrates AGEs increased apoptosis of ADSCs via a RAGE-p38 MAPK-mediated pathway. Together with other related studies, these results could provide insights about how to block the adverse effects of AGEs on ADSCs, and could lead to improvements in the clinical application of ADSCs.

## Materials and methods

### Cell culture

This study was conducted in accordance with the ethical standards laid out in the Declaration of Helsinki (1975) and was approved by the Institutional Ethics Committee at China Medical University. Adipose tissue samples were obtained with informed consent from patients at Shengjing Hospital. The ADSCs were isolated and harvested as previously described [24]. Briefly, adipose tissues were digested with type I collagenase (Roche Diagnostic, Mannheim, Germany) under gentle agitation at 37°C for 30 min. The enzyme activity was neutralized with FBS, and the suspensions were centrifuged at 300 *g* for 10 min to remove floating mature adipocytes. The cells were seeded into DMEM medium supplemented with 10% FBS. The cultures were maintained at 37°C in a 5% CO_2_ incubator. After 48 h, non-adherent cells were removed, and adherent cells were washed 3 times with phosphate-buffered saline (PBS). The medium was changed every 3 days, and the cells were subcultured when the monolayer of adherent cells reached confluence. ADSCs were used in experiments between passages 3–6.

### Cell identification

Flow cytometry was used to detect cell surface markers on the cultured cells. The cells were trypsinized with 0.05% trypsin-EDTA, collected in regular mesenchymal cell media, and centrifuged for 5 min at 800 *g* at 4°C. Then, the cells were resuspended in PBS containing 1% bovine serum albumin at 10^6^ cells/ml and incubated with monoclonal PE-conjugated antibodies for CD45, CD34, CD14, and HLA-DR; an FITC-conjugated antibodies for CD90 and CD105 or matching isotype controls (all from Becton-Dickinson, USA) for 45 min on ice. The cells were subsequently washed with PBS, fixed with 1% formaldehyde, and analyzed by flow cytometry. A minimum of 10,000 cell events per sample were acquired on a FACSCalibur flow cytometer.

### Treatment with specific inhibitors

For some experiments, specific inhibitors were added to cells and incubated for 60 min before treatment with AGE-HSA (St. Louis, USA). The ERK inhibitor (U0126), JNK inhibitor (SB203580), and p38 inhibitor (SP600125) were purchased from Santa Cruz Biotechnology (Santa Cruz, CA, USA).

### Small interfering RNA transfection and contransfection

Cells were plated in 6 cm^2^ dishes (4 × 10^5^ cells per dish). The cells were grown to 70% confluence and transfected with siRNA complexes or transfection reagents alone (mock transfection) using Lipofectamine 2000 (Gibco BRL) according to manufacturer’s instructions as previously described [29]. A scramble siRNA duplex (fluorescein) was used as a negative control. In the rescue condition, subjects were cotransfected with siRNA and a *PCDNA3.1-RAGE* expression construct.The final concentration of siRNA used for each transfection was 50 nM.

### Apoptosis assay

For the apoptosis assays, cells were plated in 96-well plates at a density of 2.0 × 10^4^ cells per well. They were incubated until they were approximately 80% confluent, and then they were changed to the assay medium. To induce apoptosis, ADSCs were exposed to HSA (300 μg/ml) or AGE-HSA at various concentrations (50-500 μg/ml) for 24 h. To determine the role of different signaling pathways in apoptosis, ADSCs were pretreated with MAPK inhibitors U0126(50 μmol/l), SB203580(10 μmol/l), SP600125 (50 μmol/l), or RAGE siRNA for 60 min where indicated, and then apoptosis was induced with 300 μg/ml AGE for 24 h. The level of apoptosis was determined using the Cell Death Detection ELISAPLUS kit (Roche Applied Science, Indianapolis, IN), which detects cytoplasmic histone-associated DNA fragments, according to the manufacturer’s instructions as previously reported [4]. Absorbance was measured at 405 nm with a PowerWave 2 multiplate reader spectrophotometer.

### Caspase-3 activity assay

As a marker of apoptosis, Caspase-3 activation was assessed using the Caspase-Glo3/7 Assays (Promega, Madison, USA). Cells were plated in 96-well plates at a density of 2.0 × 10^4^ cells per well and incubated for 24 h. The cells were treated as described above in the apoptosis assay. After the cells were treated, 60 μl of the supernatant was aliquoted from each well to a new 96-well plate. An equal volume of Caspase-Glo 3/7 reagents were added, and then incubated for 1 h at room temperature before measuring the luminescence of the samples.

### RNA extraction and reverse transcription-polymerase chain reaction (RT-PCR)

RT-PCR was performed as previously described [24]. Briefly, total RNA was extracted from each sample using Trizol reagent (Invitrogen) according to the manufacturer’s instructions, and dissolved in DEPC-treated water. The quality of the isolated RNA was checked by agarose gel electrophoresis, and RNA concentration was determined by measuring optical density at 260 and 280 nm. Total RNA (2 μg) in a final volume of 40 μl was subjected to reverse transcription. cDNA synthesis was carried out using random hexamer primers and MMLV reverse transcriptase, under the conditions recommended by the manufacturer (Invitrogen). Specific primers were manually designed using Gene Runner software (Hastings Software, Inc.). Sequences of primers and PCR conditions used are shown in Table 2.

**Table 2:** **Sequences of primers and PCR conditions**

	Primer sequence	Product size (bp)	Ta (°C)/cycles
**RAGE**	**S: 5′-CTGGTGTTCCCAATAAGG-3′**	**372**	**58.5/38**
	**AS: 5′-AGGTCAGGGTTACGGTT-3′**		
**GAPDH**	**S: 5′-ACCACAGTCCATGCCATCAC-3′**	**452**	**56.0/26**
	**AS: 5′-TCCACCACCCTGTTGCTGTA-3′**		

S, sense; AS, antisense; Ta, annealing temperature.

### Plasmid construction

ADSCs were lysed with isogen reagent (Nippon Gene, Tokyo, Japan) for RNA extraction. Total RNAs were reverse transcribed with SuperScript II Reverse Transcriptase (Takara) into cDNA. The full length human RAGE CDS sequence was amplified by PCR and specific primers with kpn1 restriction sites were designed according to sequence number NM_001206954.1 on (http://www.ncbi.nlm.nih.gov ). Full length RAGE was inserted into a pCDNA3.1 vector to construct a mammalian expression plasmid (PCDNA3.1-RAGE). Successful insertion was confirmed by sequencing.

### Western blot analysis

Protein was isolated from harvested cells using mechanical disruption and the Mammalian Cell Lysis Kit (Sigma-Aldrich, St Louis, MO) according to the manufacturer’s instruction. The proteins were separated on 1 mm NuPage Novex 10% Bis-Tris gels using the NuPage MOPS SDS Buffer Kit (Life Technologies, Carlsbad, CA, USA) followed by electrotransfer to 0.2 mm nitrocellulose membranes (Pall, Port Washington, WI, USA). Nonspecific binding sites were blocked with 5% bovine serum albumin in PBS for 1 hour at room temperature (RT). Membranes were then incubated with diluted primary antibody (1:1000; Cell Signaling Technology, Inc.) at 4°C overnight. Following three washes with PBS containing 0.5% Tween-20, membranes were incubated with diluted secondary antibody (GE Healthcare, Buckinghamshire, UK) at RT for two hours. The signal was visualized with an enhanced chemiluminescent reagent (Amersham Biosciences, Piscataway, NJ). For the protein loading control, blots were stripped and stained for GAPDH using an anti-GAPDH antibody (1:2000, AbCam, Cambridge, MA).

### Statistical analysis

Statistical analysis was performed using SPSS version 19.0 software. Data are presented as means ± standard deviation (SD). Univariate comparisons of the means were performed using the Student’s t test with a significance threshold of p < 0.05. The data shown in the figures are representative of three independent experiments.

## Abbreviations

AGE-HSA: Advanced glycation end products-human serum albumin
ADSCs: Adipose tissue-derived stem cells
RAGE: Receptor for advanced glycation end products
MAPK: Mitogen-activated protein kinase
EPC: Endothelial progenitor cell
JNK: C jun N terminal kinase
RT-PCR: Reverse transcription-polymerase chain reaction.
